# Uncovering hidden pathways: structural brain networks under connected speech in post-stroke aphasia

**DOI:** 10.1093/braincomms/fcag260

**Published:** 2026-07-07

**Authors:** Ping-Jung Duh, Ioana Sederias, Vitor C Zimmerer, Haya Akkad, Alexander P Leff, Thomas Hope, Jenny Crinion

**Affiliations:** Institute of Cognitive Neuroscience, University College London, London WC1N 3AZ, United Kingdom; Institute of Cognitive Neuroscience, University College London, London WC1N 3AZ, United Kingdom; Department of Clinical Neurosciences, University of Cambridge, Cambridge CB2 0SZ, United Kingdom; Department of Language and Cognition, University College London, London WC1N 1PF, United Kingdom; Institute of Cognitive Neuroscience, University College London, London WC1N 3AZ, United Kingdom; Institute of Cognitive Neuroscience, University College London, London WC1N 3AZ, United Kingdom; Wellcome Centre for Human Neuroimaging, University College London, London WC1N 3BG, United Kingdom; Wellcome Centre for Human Neuroimaging, University College London, London WC1N 3BG, United Kingdom; Institute of Cognitive Neuroscience, University College London, London WC1N 3AZ, United Kingdom; Wellcome Centre for Human Neuroimaging, University College London, London WC1N 3BG, United Kingdom

**Keywords:** lesion, speech, multiple-demand, imaging methodology

## Abstract

Individuals with post-stroke aphasia, an acquired language disorder, face significant communication challenges essential for daily life. Surprisingly, little is known about how focal brain damage disrupts the bilateral anatomical integration of language and multiple-demand brain networks required for higher level, connected spoken language following aphasic stroke. To address this, we investigated the anatomical network correlates of spoken language abilities in a selective subgroup of thirty-six individuals with chronic post-stroke aphasia who had preserved single-word comprehension and monosyllabic word repetition (mean age 59 ± 12.51 years; 26 males/10 females) using an innovative methodological framework. Employing a lesion quantification toolkit and graph theory analyses of T1 volumetric MRI brain scans we measured individual’s brain structural network efficiency. We then quantified the efficiency of their spoken language abilities using measures of bigram frequency, collocation, and speech connectivity using a frequency language analysis tool. Combining these brain and behavioural data, we found that higher structural efficiency in bilateral language networks significantly correlated with better connected speech abilities. By quantifying the impact of focal lesions on not only the left (dominant) language network but also bilateral language and multiple-demand networks, we were able to account for variance in aphasic’s higher level, connected speech abilities. *Post hoc* analyses showed (i) word-level spoken language behaviours were associated with discrete left temporo-parietal, using voxel-based correlational methodology; (ii) while bilateral language and multiple-demand structural network efficiency was primarily sensitive to higher-level language behaviours, loading additional brain–behaviour variance beyond distributed voxels. These findings replicate prior research on word-level language behaviours and extend our insights into how bilateral brain networks are integrated in connected spoken language. Taken together, our findings illustrate how connected speech abilities, beyond the single-word level, in post-stroke aphasia rely on distributed bilateral anatomical networks. By utilizing widely available structural MRI brain scans alongside connected speech analyses mirroring more closely real-life speech communication, the framework we propose here offers a clinically accessible approach to enhance aphasia research and treatment. By focusing on structural network efficiency, it provides a transformative method to better understand the relationship between brain anatomical connectivity and spoken language skills, potentially guiding more effective aphasia interventions.

## Teaser

Duh *et al*. examined how brain damage disrupts networks in 36 post-stroke patients with aphasia. Using structural MRI and speech analysis, they found that connected speech was particularly sensitive to structural network efficiency across both hemispheres. These findings point to new approaches targeting distributed bilateral networks for aphasic speech recovery.

## Graphical Abstract

**Graphical Abstract:**
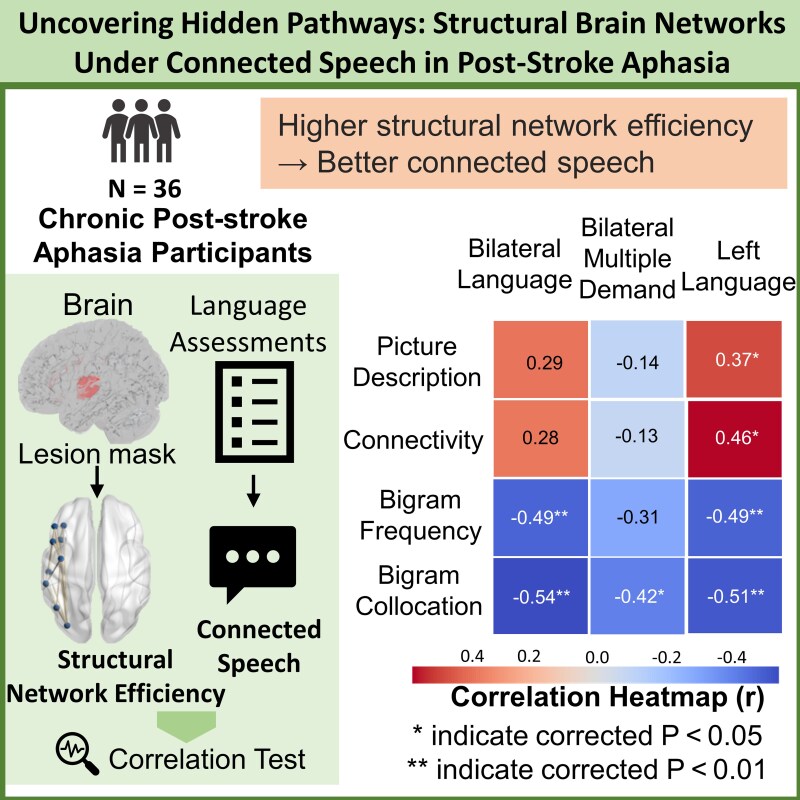

## Introduction

Aphasia is an acquired language disorder most commonly caused by a stroke affecting the dominant, typically left, hemisphere. While the lesions that cause aphasia primarily impact language-related brain regions, growing evidence indicates that post-stroke patients with aphasia (PWA) also experience impairments in other cognitive domains crucial for daily communication, such as working memory and cognitive flexibility.^1-4^ Language and non-language cognitive functions are interrelated, with evidence suggesting mutual interplay on speech communication abilities with language deficits disrupting non-language cognition performance^5^ and cognitive impairments impacting on functional language abilities.^2,6^ For example, working memory impairments often persist in PWA even after apparent recovery on standard language tests.^7^ These deficits may negatively affect everyday communication and, in some cases, limit treatment success.^8,9^

Current standardized measures of language ability, especially those used in routine clinical practice, primarily focus on word-level tasks, such as naming and single-word comprehension, which assess isolated phonological and semantic processing. Preserved performance on a naming task may not detect more subtle impairments in semantic retrieval or verbal working memory that can impact a person’s ability to communicate sequence information in conversation. While some include high-level tasks, such as sentence comprehension (requiring syntactic parsing and working memory), these measures often fail to capture the integrative cognitive-linguistic demands of real-world communication, such as those involved in naturalistic discourse.^10^ Spoken picture description tasks test the ability of the person with aphasia to understand and describe a complex picture. Though they are not a test of narrative or conversational speech, they do give an indication of the ability of the person with aphasia to produce connected speech within a constrained context. As such, they provide more naturalistic, open-ended response contexts that require the simultaneous use of multiple language and cognitive processes, such as verbal working memory, sequencing and connected speech planning.^11^

Here, we use the term connected speech to refer to sentence-level spoken language production involving multi-word sequencing and syntactic structure, as distinct from discourse- or narrative-level coherence. While some clinical assessment of PWA includes connected speech components,^12^ they are often scored using broad ratings. For example, although CAT ratings provide a structured index of performance, which are scored across syntactic variety (0–6), grammatical well-formedness (0–6), speed (0–3) and the number of information-carrying words, they are limited in sensitivity to the richness of speech data, making the task more akin to a rating-based measure than a comprehensive assessment of connected speech. A proposed alternative method for quantitative speech production analysis is the Frequency in Language Analysis Tool (FLAT),^13,14^ an automated toolbox for quantification of language-based frequentist-use of spoken language features. By characterizing connected speech samples at multiple linguistic levels, including frequency, novelty and connectivity of word combinations used, it aims to provide novel insights into more complex spoken language performance.

It appears that familiarity of expressions is an important factor for organization of language in the brain. Surprisingly, analyzing connected speech from a usage-based or frequentist perspective has rarely been explored in the context of PWA. A few studies in PWA and those with Alzheimer’s disease have reported reduced linguistic creativity during speech, relying heavily on familiar phrases and fixed expressions.^13-15^. For example, in non-fluent aphasia populations, bigram (i.e. two-word combination) frequency and collocation scores show significantly high values relative to controls, which can be associated with reduced lexical-semantic, grammatical or working memory capacity, resulting in less flexible language processing.^14^ Overreliance on familiar combinations has been regarded a compensatory mechanism to mask linguistic impairments.^16-18^.

With this interplay between language and cognitive control underpinning everyday communication behaviour, speech production likely involves coordinated activity across multiple brain regions, i.e. both language-specific as well as multiple-demand (MD) regions in both hemispheres.^19,20^ For most people, the core language network is left-lateralized relying on areas such as left inferior frontal gyrus and left superior temporal gyrus.^21,22^ However, additional cognitive functions including holding the conversation topic in mind, organizing ideas sequentially, and flexibly shifting between themes likely engage bilateral regions classically implicated in non-verbal executive functions and working memory, such as the dorsolateral prefrontal and parietal cortices.^23,24^ While left-hemisphere structures are typically dominant, residual function in contralateral and bilateral temporo-parietal areas may also support function and recovery after aphasia stroke.^25^ Consistently with this, our group found increased bilateral temporal lobe grey matter tissue density associated with improved auditory comprehension abilities in chronic PWA following behavioural training.^26^ Suggesting that preserved functional, and by association anatomical connectivity between hemispheres, may underpin language recovery after brain damage.

The complex interactions between multiple brain regions that support language recovery lead us to consider a broader network perspective in language processing. Cognitive impairments, including those involved in speech production, are often described in terms of network disruptions.^27,28^ Deficits may arise from anatomically intact but disconnected brain regions within a network.^29-32^. For example, structurally intact regions may be functionally impaired due to white matter disconnection from key network nodes. Residual anatomical connections of these regions could provide a neural basis for compensatory cognitive functions to support speech production.^33,34^

To quantify anatomical damage associated with speech deficits in aphasia, researchers have traditionally used mass univariate approaches such as voxel-based lesion analyses. This method correlates variance in structural brain damage, e.g. from a T1 volumetric MRI brain scan, with variance in PWAs’ behavioural performance.^35^ However, a limitation of the voxel-based lesion method is that it treats each voxel independently and only considers regions with direct damage, which may not fully capture the distributed network disruptions underlying complex behaviours.^32,36^ A proposed alternative and complementary approach is the examination of white matter pathways connecting various brain regions using diffusion tensor imaging^37^ or T1-based disconnectomics.^38^ While both methods provide valuable insights into structural connectivity, they primarily quantify tract-level or regional disconnection and are, therefore, less suited to capturing network-level properties such as the efficiency of information transfer across the brain’s structural network. In parallel, multivariate voxel-based mapping and related modelling approaches can capture distributed lesion–symptom relationships, yet their interpretation is often not straightforward, as they may yield complex spatial patterns that require *post hoc* validation. Given these challenges, we adopted a graph-theoretical framework, estimating structural network efficiency (SNE) to quantify how efficiently information is transmitted across the brain’s structural network. This provides a transparent and theoretically grounded measure of large-scale network organization.^39^

To understand brain and behaviour function primarily in the healthy brain, many groups have used functional regions of interest (fROI) defined by task-based fMRI activation patterns to parcellate language and MD cognitive networks,^40,41^ derived from the large openly available cognitive and language localizer dataset. In PWA, how residual anatomical connectivity between these group-based fROIs, assessed using T1 volumetric brain scans, relates to speech abilities remains unclear. While group-based fROIs do not account for individual variability in functional anatomy, they provide a consistent framework for linking structural damage to behavioural outcomes across participants. Applying graph-theoretical measures of structural network efficiency (SNE) within these fROIs^42^ helps quantify how network integrity supports language performance.

**Figure 1:**
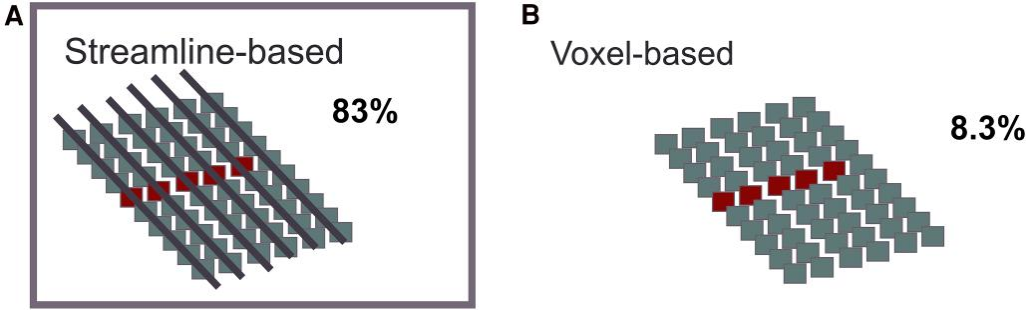
**An example illustrating the difference between streamline-based and voxel-based tract disconnection methods.** Both panels show a tract with 60 voxels, five of which are lesioned. Despite the same lesion volume, the two methods yield different disconnection estimates. (**A**) the streamline-based method (showed 83% severity)—used in this study—calculates the proportion of streamlines disrupted by the lesion. This measure of disconnection ‘severity’ (i.e. the proportion of affected streamlines between brain regions) is derived from the Lesion Quantification Toolkit.^43^ (**B**) Voxel-based methods (showed 8.3% severity) measure the proportion of lesioned voxels in the tract.

To enhance sensitivity to disconnection, the lesion quantification^43^ approach employs streamline-based tract disconnection severity, rather than voxel-wise tract lesion load.^44^ Tract lesion load estimates the proportion of damaged voxels within each tract, but it does not account for spatial distribution of lesions across streamlines. Thus, a small lesion volume could still substantially disrupt connectivity if distributed across streamlines (see Fig. 1 in Materials and methods section). The approach thereby directly captures tract disconnection and may better detect network disruptions relevant for brain function.^43^

Building on this, in this paper, we examine whether the SNE approach can effectively measure the integrity of the disrupted structural network underlying speech production abilities in a group of 36 PWA. Our approach diverges from previous studies^45-47^ that looked at either whole-brain efficiency or local efficiency around a single region. Whole-brain efficiency reflects global communication capacity but lacks specificity for functional language networks^43^; while local efficiency captures regional clustering, but does not assess integration across distributed areas.^45,47^ To address these here, we explore if structural network-specific measures would better align with the functional organization of language and cognitive control systems.

To address the challenge of overfitted issues with our sample size, we reduced our brain variable dimensions, by focusing on two functional regions of interest networks (fROIs). These two fROIs were defined using the extensive database of normal healthy subjects’ functional activation patterns for language and MD cognitive tasks available from Fedorenko and colleagues.^40,41^ These fROIs were chosen to capture the integrity of networks supporting language-specific and MD cognitive processes, which are often impaired in aphasia.^2,8,48^ The language fROI includes regions consistently activated during language tasks in healthy individuals, while the cognitive fROI comprises regions engaged in various demanding cognitive tasks, such as working memory and cognitive control. We then used SNE to quantify ipsilesional (left hemisphere), contralesional (right hemisphere) and bilateral connectivity (both intrahemispheric and interhemispheric) with these same two functional brain networks. This enabled us to examine the impact of the left-hemisphere lesions in our PWA sample (compared to the normative reference) on damaged dominant language and non-dominant (contralesional) language networks and bilateral MD networks.

We hypothesized that SNE captures brain–behaviour relationships at the network level. We predicted higher SNE values of bilateral language and MD brain networks would be associated with better spoken language abilities within the PWA group, complementing the localized lesion-deficit mappings from voxel-based method. The same participant cohort has previously been described in Akkad *et al*.^49^ in relation to voxel-based lesion–symptom mapping of language and cognitive deficits. The current study addresses a distinct question, focusing on connected speech production and its relationship to network-level brain organization. We tested associations between variance in SNE and behavioural scores with bilateral, left hemisphere, and right hemisphere language and MD network measures, while controlling for lesion volume. To increase our sensitivity to connected speech production abilities, our analyses included (i) frequentist measures using FLAT analysis of PWAs’ speech samples from custom picture description tasks and (ii) anatomical measures of distributed brain network using SNE.

## Materials and methods

### Participants

**Figure 2:**
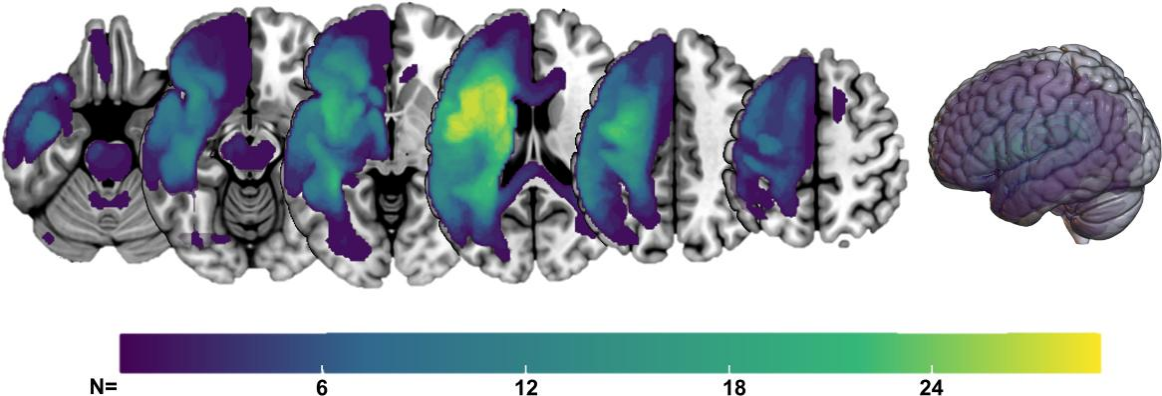
**Lesion overlap map for *N* = 36 PWA.** Colour-scale represents frequency (number of patients) of regional brain damage, with yellow indicating most frequently damaged brain regions (i.e. regions affected in >24 patients), and dark blue indicating less frequently damaged regions (<6 patients). All participants had a dominant left-hemisphere stroke resulting in aphasia; one participant also had a previous asymptomatic right-hemisphere stroke in the posterior parietal lobe. Excluding this PWA did not change the results and given they met all other recruitment criteria we include their data throughout. Results are shown overlaid on the MNI template brain, created in MRIcro GL (https://www.nitrc.org/projects/mricrogl).

A total of 36 PWA (26 males, 10 females) in the chronic post-stroke phase took part. All were native English speakers with left-hemisphere stroke at least 12 months prior to taking part in the study. The key inclusion criteria were that each participant had (i) anomia as assessed by the naming subtest of the Comprehensive Aphasia Test^50^ (CAT) and (ii) relatively good single-word comprehension as per the subtests of the CAT.^50^ (iii) Preserved repetition of monosyllabic words on the CAT,^50^ used to exclude participants with severe motor speech output deficits. At this level, repetition places minimal demands on verbal short-term memory. (iv) No evidence of speech apraxia as assessed by the Apraxia Battery for Adults.^51^ All had good functional hearing and visual acuity, no prior neurological or psychiatric disorders and no contraindications for MRI. The lesion overlap map illustrates the PWAs’ pattern of left-hemisphere brain damage (Fig. 2). The PWAs’ descriptive statistics of demographic are described in Table 1 and the overall clinical data can be found in Supplementary Table 5. Participants were recruited at University College London and the study was approved by the Central London Research Ethics Committee, UK. The current sample includes the same PWA as reported in in the study of Akkad *et al.*^49^ that used voxel-based correlational methodology (VBCM) to investigate the neural correlates of language and domain-general cognitive deficits. Inclusion criteria required preserved mono-syllable single-word repetition abilities and absence of speech apraxia to minimize potential confounds from severe motor speech impairments; and good single-word comprehension to ensure they understood the task demands. The present study extends prior work by investing how network-level brain measurements relate to linguistic performance in connected speech.

**Table 1:** Thirty-six PWA descriptive statistics of demographic, clinical data and behaviour test scores

		Mean	SD	Range (min—max)
*Demographic*				
Age (years)		59	12.51	29–82
Education (years)		13.8	2.38	10–17
Time post-stroke (years)		10.36	8	1–34
Lesion volume (cm^3^)		131.7	79.89	1.51–403.11
*Behavioural performance*				
CAT spoken comprehension	Words	26.75	3.08	17–30
	Sentences	22.5	5.34	13–32
CAT speech production	Naming	35.11	7.47	13–48
	Picture description	19.56	10.21	1–40
FLAT scores of custom picture description	Bigram frequency	1624.5	620.19	204–3620
	Bigram collocation	12.65	4.19	1–21
	Connectivity	0.46	0.15	0.05–0.69

The CAT picture description score refers to the standard speech production measure from the CAT,^50^ which is based on ratings of speech rate, grammatical well-formedness, and the number of informative words. The overall data of each participant can be found in Supplementary Tables 5 and 6.

### Behavioural data

All PWA completed a comprehensive assessment of spoken language abilities. This included spoken language production and comprehension assessments via subtests of the comprehensive aphasia test battery,^50^ as well as custom-made spoken picture description tasks. The four custom-designed images each comprised scenes with 100 high-frequency, high-imageability English lexical items. Images were more complex compared to the composite picture description scene of the CAT. While we retain the CAT picture description as a clinical benchmark, the custom tasks were suited for probing sentence-level connected speech production abilities. Items were often not causally related, which places additional demands on lexical selection, sequencing and sentence construction rather than narrative organization. Details of custom picture description tasks can be found in Supplementary Figs 1–4 and Supplementary Tables 1–4.

### Connected speech—FLAT frequency, collocation and connectivity score

To quantify PWAs’ connected speech performance, linguistic measures were computed utilizing the Frequency Language Analysis Tool^18^ (FLAT). FLAT is a software program that quantifies usage-frequency based language variables for word combinations. It extracts frequencies by looking up each unit within transcriptions from spoken language in the British National Corpus, which represents everyday communication. FLAT values cover several distinct linguistic dimensions such as frequency, novelty and connectivity. Here, we focused on the frequency of unique bigrams (i.e. two-word combinations, which only appear once per sample—no repetition). Lower frequency word combinations generally indicate greater lexical capacity.^17^

The novelty dimension reflects the linguistic creativity within the sample, measured by collocation strength. Collocation strength refers to the occurrence of two words together relative to how often each individual word appears. Collocation strength is computed as bigram *t*-scores. Stronger collocations are likely processed in a holistic, formulaic manner^17^ (i.e. retrieved as one unit). Formulaic language poses fewer demands to lexical and syntactic processes.^14^ Overreliance on formulaic language can therefore suggest a diminished capacity to generate rare or novel expressions,^52^ which hinders communication.

FLAT measures connectivity of language production by computing the proportion of words occurring in grammatical trigrams (rather than single-word or two-word chunks). The ability to produce higher proportions of grammatically meaningful word sequences reflects stronger skills in sentence formulation and syntactic processing.^13,18^ The descriptive statistics of PWA on standardized tests of CAT subtests and FLAT scores of custom picture description can be found in Table 1. The complete test scores and healthy subjects’ performance data are available for reference in Supplementary Tables 6–8.

### MRI data processing—lesion quantification

Structural T1-weighted whole-brain MRI scans were acquired on a 3T Siemens TIM-Trio system at the Wellcome Centre for Human Neuroimaging. Images were registered into standard Montreal Neurological Institute (MNI) space using a modified unified segmentation–normalization procedure.^53^ Images were smoothed with an 8 mm full-width at half-maximum (FWHM) Gaussian kernel. To match anatomical parcellations for later lesion quantification measurements, the lesion masks were binarized from these images, re-sliced to 1 mm isotropic voxel dimension and re-sampled to image dimensions of 181 × 217 × 181 in nifti file format. All pre-processed procedures were computed within Statistical Parametric Mapping software (SPM 12) running under MATLAB 2022a. The segmented lesion masks were then used as inputs in the Lesion Quantification Toolkit (LQT),^43^ which estimates the proportion of streamlines between brain regions based on lesion–tract intersections.

### MRI data—SNE

**Figure 3:**
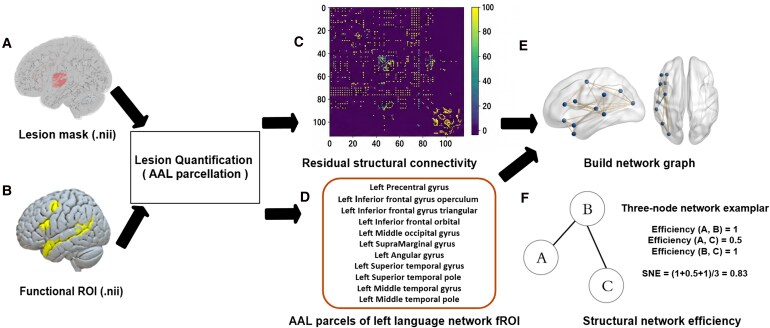
**Pipeline to estimate SNE in functionally-defined brain networks for each individual.** (**A**) Each PWA’s lesion mask is entered as an input to the lesion quantification toolkit.^43^ (**B**) A functional network ROI is input into the quantification toolbox to calculate the overlapping brain regions in the AAL structural parcellation. Steps A and B are generated in SurfIce software. (**C**) As a result from step A, each lesion mask generates a residual structural connectivity matrix. (**D**) The calculated result from step B identifies the AAL parcels found within the functional ROI. (**E**) A graph is constructed by combining the connectivity matrix and selected ROIs together. The edges were binarized at 70% threshold to form the graph. The brain visualization is created in BrainNetViewer (https://www.nitrc.org/projects/bnv). (**F**) A SNE measure can be calculated as demonstrated in a three-node network exemplar. Structural efficiency between nodes: A and B and between nodes: B and C are both 1 because there are direct paths between them. For node A and node C, there is no direct path between them. The shortest path from node A to node C is to go through node B first, therefore, the total path value is 2. The efficiency between them is the inverted value of this path, which is 0.5. The overall SNE is the average of all efficiency values in the network, which in this exemplar is 0.83. AAL, Automated Anatomical Labeling parcellations^54^; SNE, structural network efficiency; ROI, regions of interest.

To investigate the relationship between the lesioned brain’s residual structural connectivity and PWAs’ speech production performance, we used SNE to quantify the connectivity between regions within two functional brain networks supporting language-specific and MD cognitive processes. The two functional brain networks were selected based on the work by Fedorenko and colleagues.^40,41^ The data processing pipeline is outlined in Fig. 3. First, the structural connectivity matrices were derived from lesion and functional regions of interest (fROI) masks (see Fig. 3A and B) using Automated Anatomical Labeling (AAL) parcellations^54^ and HCP-842 tractography atlas^55^ in the LQT.^43^ The AAL parcellation offers a well-established and widely adopted anatomical parcellation scheme,^46,55-57^ facilitating comparison across studies and promoting reproducibility. With 116 cortical regions, it provides a reasonable balance between anatomical detail and computational feasibility. The HCP-842 tractography atlas was chosen for its high-resolution and extensive coverage of white matter pathways.^43,55^ To maintain consistency with Fedorenko’s lab’s fROI definitions while working in AAL space, we quantified the fROIs on AAL parcels, incorporating all overlapping regions except those with minimal overlap (less than 10% of the total area), chosen to balance inclusion of functionally relevant areas and reduce noise from marginally overlapping parcels. Details of AAL fROI nodes and their corresponding proportions can be found in Supplementary Fig. 5 and Supplementary Table 9.

The residual structural connectivity matrix comprised intact white matter connections expressed as a percentage of remaining connectivity between AAL parcels after accounting for each participant’s lesion. This was calculated using LQT that embeds lesions into the HCP-842 tractography atlas, capturing disconnection severity by estimating the proportion of streamlines disrupted by each lesion (Fig. 1), that inherently accounts for disconnection severity without excluding lesioned regions (Fig. 3C). For further technical details regarding the LQT methodological process we direct the interested readers to Griffis *et al*.’s study.^43^ After constructing the residual structural connectivity matrices, brain structural parcellations were designated as nodes, with white matter connections exceeding a 70% threshold binarized to one and utilized as edges. The threshold was selected based on prior studies^43,58,59^ as it balances network sparsity, reducing weak or spurious links while preserving the brain network’s core structure. Nodes and edges within functional regions of interest (fROIs) formed network graphs (Fig. 3D and E). The shortest path is the minimum structural connection(s) needing traversal from one region to another. Graph theory defines efficiency as the inverted shortest path length between two nodes.^42^ Thus, efficiency ranges from zero to one, with higher values indicating greater efficiency. We implemented the breadth-first search algorithm^56^ to calculate shortest path. Structural network efficiency (SNE) was defined as the mean efficiency across all nodes within the fROI. Figure 3F illustrates a graph including three nodes and its SNE.

The residual structural connectivity matrix was calculated by Lesion Quantification Toolkit^43^ running under MATLAB 2019b and the SNE was measured by Networkx package running under Python 3.9 (see Supplementary S4). Behavioural materials and additional resources are available in the Supplementary Materials.

### Statistical analysis—Spearman’s correlation

To investigate whether higher residual SNE within fROIs correlated with better preserved behavioural functions in PWA at the group level, we examined the correlation between individual behavioural scores and SNE in both lateralized and bilateral fROIs. Given the PWA brain data did not always satisfy the assumption of normal distribution we used two-tailed Spearman’s rank correlation coefficients consistent with prior brain–behaviour mapping studies.^2,60^ For greater lesion-behaviour statistical power, we used *P*-value correction based on 10 000 random permutation tests following network-based stats^59^ and reported at *P* < 0.05 and *P* < 0.01 alpha threshold. Lesion volume, estimated by the automated lesion identification method,^53^ was included as a covariate in the statistical models.^30^ Other demographic variables were not included, as they showed no consistent effect on language outcomes. Analyses were run with SciPy under Python 3.9.

## Results

### Brain network correlations with connected (narrative) speech production

**Figure 4:**
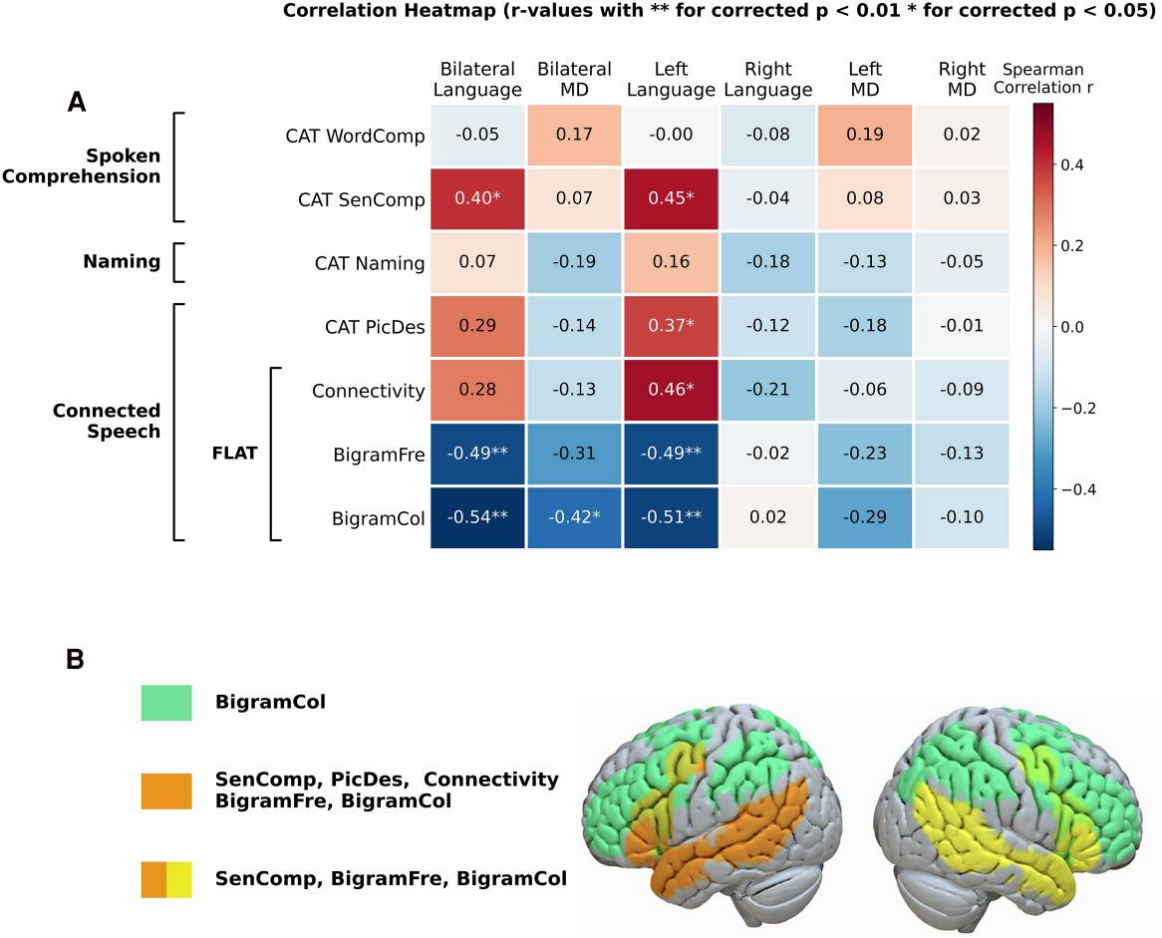
**Correlations between structural network efficiency (SNE) and speech production performance in PWA.** (**A**) Heatmap shows Spearman’s correlation coefficients (R) Between structural network efficiency measures and behavioural scores. Colour indicates correlation strength and direction (red = positive, blue = negative) for bilateral, left hemisphere, and right hemisphere language and MD networks, with darker shades representing stronger correlations. Significance was determined using Spearman’s correlation across *N* = 36 subjects, with *P*-values corrected for multiple comparisons via permutation testing (100 000 permutations). Values marked with * indicate corrected *P* < 0.05; ** indicate corrected *P* < 0.01. (**B**) Visualization of brain–behaviour correlations overlaid on cortical surface using SurfIce software, showing significant correlations. Right: functional networks of interest Brain rendering: Visualization of overlaid correlations in SurfIce (green—bilateral MD networks; orange—left language network; yellow and orange—bilateral language networks); Left: a visual representation of how SNE in each functional network relates to distinct measures of connected speech. WordComp, word comprehension; SenComp, sentence comprehension; PicDes, picture description; BigramFre, bigram frequency; BigramCol , bigram collocation.

In this group of PWA, better performance on connected speech measures was associated with greater structural efficiency in the left language network. Specifically, FLAT connectivity measure (*r* = 0.46, *P* = 0.013) and CAT standardized picture description scores (*r* = 0.37, *P* = 0.029) showed positive correlations with left-hemisphere language network SNE. Complementing these findings, FLAT measures of bigram frequency and bigram collocation—where lower scores reflects more creative, less formulaic language—were correlated with higher SNE in both left and bilateral language networks (*r* = −0.49, *P* = 0.003 and *r* = −0.54, *P* = 0.001, respectively). The FLAT bigram collocation performance measure was the only one negatively associated with bilateral SNE measures of the MD network (*r* = −0.42, *P* = 0.013). See Fig. 4 for full reported results.

### Brain networks correlate with sentence tasks but not word-level speech production

Our analysis revealed a significant relationship between SNE and language behaviours at high-level but not at word-level language behaviours.

There was a significant positive correlation between SNE of the bilateral language network and spoken sentence comprehension (*r* = 0.40, *P* = 0.018) in PWAs. The increased efficiency within residual bilateral hemisphere structural language network was associated with better spoken sentence comprehension. In contrast, word-level language processing including naming and spoken word comprehension did not show significant correlations with SNE in any of the assessed networks.

### *Post hoc* 1: voxel-based lesion–symptom mapping

To investigate whether word-level language behaviours could be associated with focal lesions in specific brain regions, we conducted a *post hoc* voxel-based correlational methodology^49,61^ (VBCM), a variant of univariate voxel-based lesion–symptom mapping^35^ (VLSM), using the same behavioural measures. A voxel-wise *F*-test was used to compare the fuzzy lesion status between patients with their performance on each neuropsychological assessment. Total lesion volume was included as a covariate in the general linear model. Results are reported at *P* ≤ 0.001 voxel-level and *P* < 0.05 family-wise error (FWE) corrected at cluster-level, in line with previous VLSM aphasia studies^2^ and consistent with our prior work.^49^ All anatomical labels were based on the AAL atlas in MNI space. The analyses were run using SPM 12 with MATLAB 2022b.

**Figure 5:**
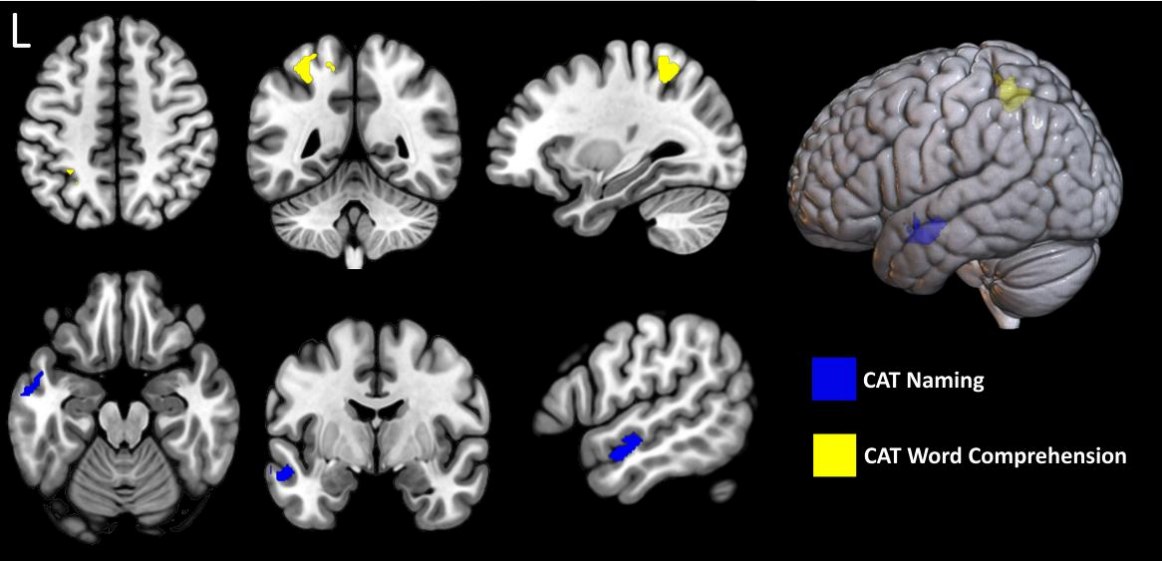
**Neural correlates of word-level speech production deficits identified by VBCM.** Results are shown at *P* ≤ 0.001 voxel-level, *P* < 0.05 FWE corrected at cluster-level. Colours indicate regions where grey matter damage correlates with impairments in different word-level speech production processes and sentence repetition: blue = naming deficits, yellow = spoken word comprehension deficits. Results are overlaid on a rendered brain template (MRIcro-GL). L denotes the left hemisphere. CAT refers to the comprehensive aphasia test.^50^

The results revealed neural correlates with word-level speech production deficits (Fig. 5 and Table 2). Damage to left middle temporal gyrus was correlated with impairments in naming. Lesions in the left superior parietal gyrus were associated with deficits in spoken word comprehension. No significant neural correlates were found for sentence comprehension and connected speech measures. The results complement our main findings by demonstrating that while word-level language behaviours do not show a significant relationship with SNE measures, they are associated with focal lesions in specific brain regions.

**Table 2:** Neural correlates of word-level speech production deficits

Behaviour assessments	Brain location	Extend voxels	*Z*	MNI coordinate		
				*x*	*y*	*z*
CAT naming	Left middle					
	temporal gyrus	149	3.31	−58	−8	−20
CAT word comprehension	Left superior					
	parietal gyrus	240	3.14	−26	−56	48

Only clusters with cluster-level FWE *P* < 0.05 are shown. Coordinates are in MNI space.

### *Post hoc* 2: canonical variate analyses

To investigate whether SNE was primarily sensitive to high-level language behaviours and provided additional brain–behaviour information beyond distributed voxels *per se*, we conducted a *post hoc* canonical variate analysis^62^ (CVA) in SPM12. CVA is a multivariate method that identifies linear combinations of variables that best explain variance in dependent variables. Here, CVA was used to quantify how much language behaviour variance could be accounted for by brain measures (SNE and voxels). The resulting canonical variates, ranked by most explained shared variance, represent dimensions of maximal covariation between the brain measures and language behaviours. Chi-square tests indicate the statistical significance of the amount of shared variance that can be explained.

**Figure 6:**
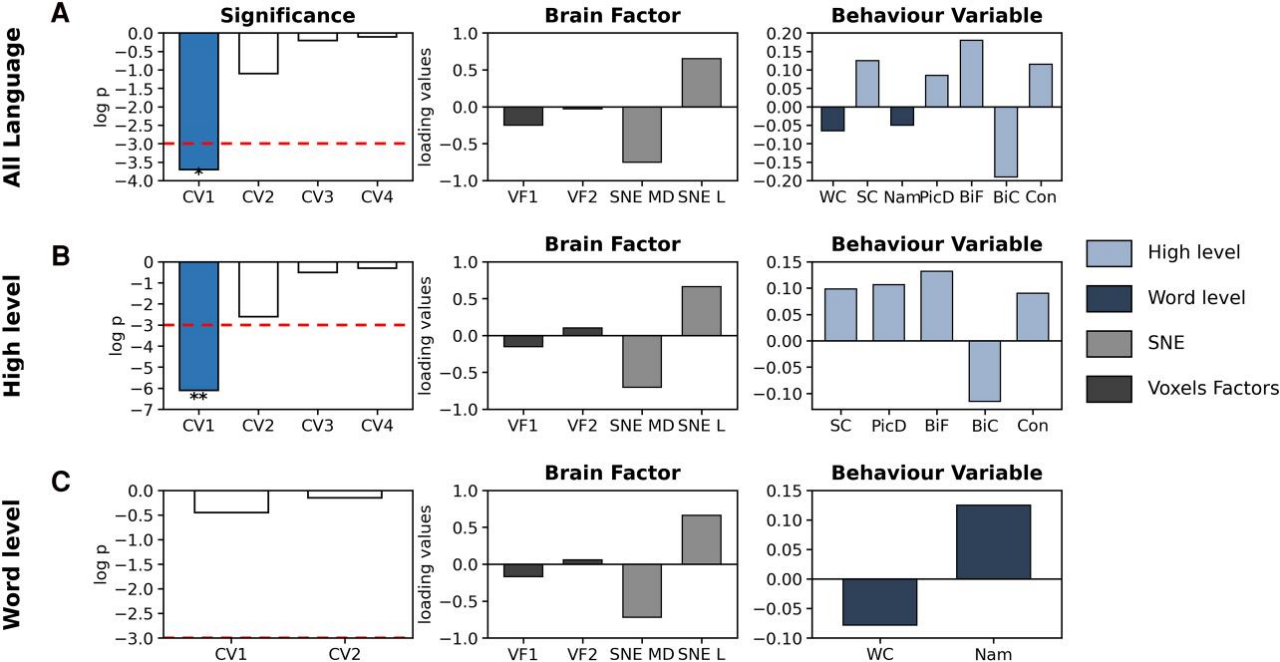
**CVA results: SNE and voxels with behaviour variables at different levels.** This figure presents CVA results on *N* = 36 participants to assess relationships between brain factors (SNE and voxel-wise) and language behaviours at three levels: **(A)** all language behaviours, **(B)** high-level, and **(C)** word-level, illustrating the variable loadings and which combinations show statistically significant associations. Each row represents a behaviour level, with columns showing: **Significance** log *P*-values from chi-squared tests for canonical variates (CVs) (with * *P* < 0.05, ** *P* < 0.01); **brain factor** loadings on the first canonical variate (CV1); and **behavioural variable** loadings to CV1. Significant associations were found for all behaviours (χ^2^ = 45.1, df = 28, *P* = 0.021) and high-level behaviours (χ^2^ = 43.0, df = 20, *P* = 0.0021), both showing stronger loadings on SNE than voxel factors. The word-level behaviour model was not significant (χ^2^ = 5.5, df = 8, *P* = 0.708). CVA, canonical variate analysis; CV, canonical variate; VF, voxels factor; SNE, structural network efficiency; SNE MD, SNE multiple demand; SNE L, SNE language; WC, word comprehension; SC, sentence comprehension; Nam, naming; PicD, picture description; BiF, bigram frequency; BiC, bigram collocation; Con, connectivity.

First, singular value decomposition (SVD) was employed to reduce all voxels in language and MD ROIs to two factors, making them comparable to the two bilateral SNE measures. All variables were standardized to address scale differences. We performed three CVAs to examine reduced voxel factors plus SNE and language behaviours at different levels (Fig. 6).

The first CVA (Fig. 6, first row) comprised of SNE and voxel factors with all language behaviours, which reached statistical significance (χ^2^ = 45.1, df = 28, *P* = 0.021), was predominantly loaded on SNE and high-level language behaviours. The second CVA (Fig. 6, second row) comprised SNE and voxel factors with high-level language behaviours. The result revealed a significant relationship (χ^2^ = 43, df = 20, *P* = 0.0021), with SNE showing higher loading than voxel factors. The third CVA (Fig. 6, third row) with SNE and voxel factors together with low-level language behaviours did not yield significant results (χ^2^ = 5.5, df = 8, *P* = 0.708).

SNE analyses revealed significant relationships between bilateral language and MD networks and spoken language abilities. Two *post hoc* analyses utilizing (i) VBCM, identified focal left-hemisphere lesion correlates of single-word processing deficits; and (ii) CVA, demonstrated that SNE bilateral network measures were more sensitive in capturing higher-level connected language behaviours than word-level behaviours.

## Discussion

Our study aimed to investigate the relationship between SNE measures in bilateral language and MD networks and connected speech in PWA. Complementing localized lesion-deficit mappings derived from voxel-based methods we predicted a correlation between SNE measures in bilateral language and MD brain networks and spoken language abilities in PWA. The connected speech measure, a bigram collocation, was associated with bilateral language and MD SNE brain measures. In line with this result, sentence-level speech comprehension abilities and connected speech bigram frequency correlated with bilateral language SNE measures. These ‘higher-level’ speech production and comprehension abilities were demonstrated using SNE, as opposed to classic voxel-based methods, suggesting that network-based approaches may have a greater sensitivity when mapping more complex language and cognitive processes which may be more widely distributed in the damaged brain. This provides a valuable complement to the more focal, often word-level behavioural associations, established using VLSM to date.

### Connected speech production in language and MD networks

Closer inspection of PWA’s residual SNE brain measures and their speech performance found negative correlations between bilateral SNE measures and FLAT measures of bigram frequency and collocation. The negative association between bigram frequency and SNE measures in bilateral language networks may suggest that generating less frequent lexical sequences imposes greater processing demands, potentially eliciting increased engagement or plasticity within these networks.^63^ Additionally, the observed correlation between bigram collocation in bilateral MD networks raises the possibility that producing less typical word pairings draws more heavily on MD cognitive resources, such as controlled retrieval or selection. This difference hints that bigram frequency may predominantly reflect demands on language-specific systems, whereas bigram collocation might index broader cognitive control processes. However, these interpretations remain tentative and would benefit from replication and further mechanistic investigation.

That the right hemisphere has a role to play in spoken language production is not new. Previous groups have proposed its role in maintaining connections between less semantically overlapping concepts and in processing less formulaic language.^15^ For PWA, given their language impairments and left-hemisphere damage, compensatory mechanisms may involve increased engagement of right-hemisphere language regions.^64^ Our bilateral SNE findings align with task-based and resting-state functional connectivity studies showing increased bilateral and interhemispheric recruitment in PWA,^28,65^ suggesting that SNE may reflect efficient anatomical paths supporting more capacity for such functional reorganization. Our future work will test direct links between SNE and FC efficiency in a larger PWA cohort.^66^

The association of MD networks^40^ with producing novel word combinations (as indexed by bigram collocation) suggests that this sample of PWA does likely recruit additional cognitive resources/networks to support flexible and creative spoken language use.^13^ While the role of the MD network in post-stroke language production is not yet fully established, our findings extend prior work by examining structural integrity during active expressive tasks—specifically overt picture description. Recent evidence points to limited MD involvement in language processing. For example, De Clercq *et al*.^67^ and Billot *et al*.^68^ reported minimal MD recruitment during language tasks in healthy adults. This addresses the need to investigate MD contributions across diverse tasks and clinical populations.

The observed MD–language association may also reflect anatomical coverage differences. Fedorenko and Thompson-Schill^40^ used a comprehension-based language task for their localizer which does not fully encompass inferior parietal regions (IPL) areas implicated in our speech production task. As noted by Fedorenko *et al*.,^69^ the language network shows regions overlap across comprehension and production, implying that our results may reflect shared representational systems rather than distinct MD contributions.^28^ Because network nodes were defined using group-level parcels, no claims can be made here regarding the MD network’s active functional engagement.

Notably, the CAT picture description scores and FLAT speech connectivity measure correlated exclusively with solely left, not bilateral, language network SNE measures. This is consistent with the core aspects of generating precise words (information-carrying units) and grammatically correct speech still relying heavily on left-lateralized language networks,^70,71^ and why lesions here result in aphasia. Reflecting on those results, when describing complex scenes, which is likely harder for PWA due to left damaged networks may recruit bilateral and MD resources to support coherent speech production. However, the specific CAT and FLAT measures used in this study primarily capture dominant linguistic features, which may explain their association here with left-lateralized rather than bilateral or MD network involvement.

### Sentence speech comprehension in bilateral language networks

In our PWA sample, efficient residual structural network connections between bilateral language regions correlated with better sentence-level comprehension as opposed to word comprehension abilities. This correlation likely reflects the increased cognitive demands associated with processing more complex linguistic structures, in line with the integration of language and working memory processes.^72^ Sentence comprehension places significant demands on working memory components, such as maintaining multiple items and serial order information.^73^ These working memory components are consistently engaged in demanding sentence-level language assessments, which require online maintenance of increasing semantic and syntactic information for comprehension.^73,74^ For PWA, to support the maintenance and manipulation of linguistic information during sentence processing, efficient working memory integration may be particularly important, to compensate for their language deficits.^8,9^

The bilateral language network templates^41^ (fROIs) that we used here are large and arguably incorporate regions also associated with verbal working memory processes.^24,73^ This may be due to the nature of the language localizer tasks used to define the templates^40,41^ or the inherent integration of verbal working memory in language processing. Supporting the latter interpretation, a recent fMRI study showed working memory demands during naturalistic speech comprehension primarily engaged the same bilateral language-template regions^75^ suggesting that such demands can be accommodated within the language network itself. This may partly explain the absence of significant associations with MD network SNE in our study. However, MD network involvement may still arise under more ambiguous comprehension conditions.^76^ The bilateral engagement may provide a mechanism for compensation from acquired brain damage in PWA, allowing the less affected hemisphere to support speech comprehension.^26^ Efficient bilateral connections potentially facilitate the exchange and integration of semantic and syntactic information, enhancing PWAs’ ability to understand meanings from complex inputs despite damage to specific language areas.

### Distributed and focal findings: SNE and voxel-based analyses

*Post hoc* analyses revealed distinct neural substrates for basic and complex language skills. Through a series of VLSM and CVA analyses, we identified differing patterns of association between brain integrity and word-level and high-level language processes. Given the methodological differences of VLSM and SNE, we employed CVA to incorporate voxel-based data (i.e. whole-brain voxels) from language and MD ROIs alongside bilateral SNE metrics. This approach enabled us to statistically assess whether SNE can explain additional variance in language outcomes beyond voxel-based measures alone.

SNE analyses were sensitive to high-level language behaviours compared to word-level ones, as evidenced by the significant high-level language CVA (*P* = 0.0021) versus the non-significant word-level language CVA (*P* = 0.708). SNE method’s particular utility appears to be in capturing distributed neural substrates associated with more complex language processes. This aligns with the notion that complex cognitive functions rely on distributed neural networks rather than isolated brain regions, echoing recent findings by Billot *et al*.^32^ on the effects of white matter disconnections in chronic PWA.

The VLSM results identified specific regions in word comprehension and naming, which align with previous literature as well as prior findings from Akkad *et al*.^49^ on the neuroanatomical basis of semantic and phonological processes.^1,2^ Unlike the higher-level language functions associated with our SNE factors, those word-level language abilities appear more closely tied to localized brain areas and focal damage.

These findings underscore the complementary nature of SNE and voxel-based approaches. While VLSM may be sensitive to focal lesion impacts on single-word level language processes, SNE captured the distributed connectivity underpinning more complex language functions such as sentence-level comprehension and connected speech production. This emerging dissociation highlights the importance of employing multiple analytical approaches in studying the neural basis of language function in the damaged brain.

### Methodological considerations

In terms of the structural organization of post-stroke brain networks, our data suggest that the efficiency of residual path connections (possibly an index of plasticity) may be critical for cognitive function. This highlights the importance of considering structural network-level properties in understanding language performance after stroke. Our analytical framework is based on T1 volumetric brain images, which are widely available and routinely collected in clinical settings, making this approach especially useful and easy to apply.

We used group-level functional ROIs (fROIs) from Fedorenko *et al*. as a proxy for network-level organization, acknowledging that these parcellations are designed to identify candidate regions from which subject-specific activations should ideally be derived. In the absence of task-based fMRI data, this approach allowed us to approximate language and MD network boundaries. However, as emphasized by Fedorenko *et al.*,^69^ true delineation of these networks requires subject-specific mapping, as neighbouring regions may differ in function. Consequently, the observed MD-related effects should be interpreted cautiously, as they may partly reflect spatial proximity or partial overlap between adjacent networks rather than direct functional contributions. Future work using production-based and subject-level localizers in PWA will be critical to refine this distinction.

Our graph-based SNE approach quantifies connectivity across anatomical combinations, generating distinct connectivity profiles despite anatomical overlap between fROIs. While we used Fedorenko’s ROIs for consistency and comparability, the framework is flexible—it can be tailored to incorporate other anatomical parcellation schemes and functional ROIs, and is generalizable to explore diverse behavioural measures based on researcher’s specific needs. Future work could also investigate the impact of different parcellation schemes and thresholding approaches on SNE analyses.

The screening procedure may have resulted in a relatively selective sample by excluding individuals with severe output disruption, including both motor speech and some phonological impairments. This reflects a methodological decision to ensure stable sentence-level spoken language production for analysis, and future work in more heterogeneous samples will be important to assess generalizability.

Clinically, our findings also emphasize the importance of assessing spoken language abilities using broader linguistic tools, such as FLAT, that integrate multiple cognitive functions and may be more sensitive to capturing more complex speech use and difficulties following aphasic stroke. We selected bigram patterns and syntactic coherence from FLAT due to their demonstrated relevance to connected speech complexity and their alignment with PWA’s typical language impairments^13^; however, it is important to note that present connected speech task targeted sentence-level production under reduced contextual constraints, rather than full discourse-level narrative coherence. CAT picture description was not analysed using FLAT in the present study; therefore, the generalizability of the observed relationships across different connected speech tasks remains to be established. Future work incorporating both approaches will be important to address this directly. Additionally, incorporating additional linguistic measures, including NLP approaches and analysis of standardized tasks like the CAT picture description will be instrumental in advancing our understanding of connected speech production and its relationship with brain network metrics.

## Conclusion

In this study of chronic PWA, good speech performance was associated with efficient residual structural brain connections across bilateral language networks. *Post hoc* analyses highlighted how SNE measures capture higher-level speech and cognitive integration, with analyses of focal lesion patterns most sensitive to word-level deficits. These results illustrate how spoken language research can be grounded in a broader approach that integrates cognitive behaviours and structural brain network theories. This framework signals a new perspective to long-term speech performance after aphasic stroke- it depends on efficient bilateral network structure and could extend beyond language. This approach could potentially serve as a tool for assessing PWAs’ brain integrity to (i) predict individual behaviours using common SNE parameters, (ii) explain individual differences via identified network sources (i.e. different functional regions), and (iii) clarify recovery patterns and treatment response. Overall, the network perspective proposed here advances our understanding of PWA, shifting emphasis from regional damage to efficient residual circuits, providing insights to future brain research in understanding language recovery mechanisms.

## Supplementary Material

fcag260_Supplementary_Data

## Data Availability

The data described in this study are available to accredited researchers from J.C.—on request. Code used for analysis, along with behavioural materials and other resources are provided in Supplementary Material S4.
