# Supplementary material for: Uncovering hidden pathways: structural brain networks under connected speech in post-stroke aphasia
Source: Brain Commun. 2026 Jul 7;8(4):fcag260. doi: 10.1093/braincomms/fcag260 (PMC13386007; doi:10.1093/braincomms/fcag260)
Supplement: fcag260_Supplementary_Data [file fcag260_supplementary_data.docx]

**Supplementary**

**S1. Materials for custom picture description tasks and patients’ data**

The stimuli were divided into 4 blocks of 100 drawings each, with a break provided after each block. The custom picture description task comprised 4 colored scene images, each containing a subset of 100 items. Supplementary Figures 1–4 present the four scene images, and representative speech transcripts elicited during the custom picture description task. Supplementary Tables 1-4 list the corresponding 100 target items in each scene. Supplementary Tables 5 and 6 provide the demographic information and standardized assessment scores for all patients.

Participants were presented with the pictures one by one and were given 5 minutes to describe what is happening in the picture using full sentences. Participants responses were audio-recorded and the audio was encoded as transcripts. These formatted transcripts served as inputs in FLAT ^1,2^.


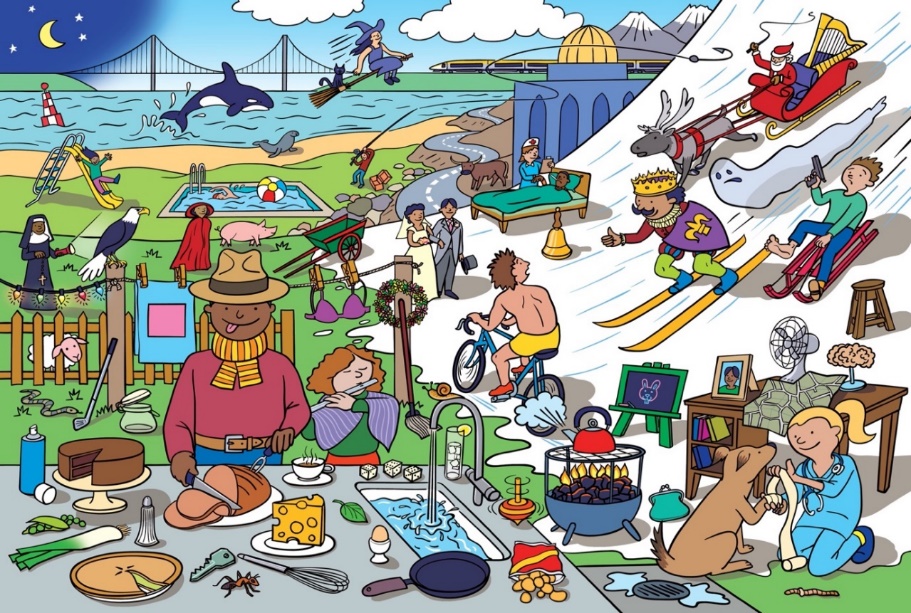


Sample example: Well. Father Christmas is coming down the slopes on Rudolf the red nose. Well. he got a red nose. Rudolf the reindeer and he's carry a harp. In front of him is the king and he got on left armour on his arm and he is. he was skiing down the slope and he's going to run into a person. bloke on a bike. Just in front of the man on the bike was the groom and bride and behind them was the doctor who's got a man in the bed with a mouthpiece. Oh. giving to. giving to him by the nurse. and she is copying what the mouthpiece said on it. Behind them. there was a cow or a bull. Now. in the distance. I can see a train which the one. and the witch on the witch's broom. and followed by a cat on the broom handle. The whale is jumping out of the sea and the. San Francisco is in the San Francisco bridge in the background. and there's a boy on the sea. And on the beach. was there was a seal. There was a boy in the lake. swimming up and down with a ball just there. there was a nun shining a torch on the eagle on. that was on the fence. Above which was a sheep which can't get his hands off the bench. There an leaning against the tree was a golf club and a snake. So. Then the at the front of the place. there was a sink Dice. Cup of tea. Cake. Egg and some meat which the guy is then cooking and by the side of the guy was the daughter.

**Supplementary Figure 1.** Picture 1 of 4 used in the custom picture description task, along with a sample script of the patient’s description.

**Supplementary Table 1.** Target words embedded in Picture 1.

| Picture 1: 100 items | | | | |
| --- | --- | --- | --- | --- |
| ant | coal | hat | pig | spray |
| back | crisps | hook | pool | square |
| ball | cup | ice | purse | steam |
| beak | desk | jar | road | stool |
| bed | dice | key | rock | swim |
| bell | dome | king | rope | tap |
| belt | drain | knee | salt | thumb |
| bike | egg | knife | scarf | toe |
| board | fan | leaf | seal | tongue |
| bra | fence | leek | shawl | top |
| brain | fire | lights | sheep | torch |
| bride | flute | map | shelves | towel |
| bull | frame | moon | shield | train |
| buoy | ghost | mop | sink | vet |
| cake | girl | nun | skis | whale |
| cape | groom | nurse | sled | whip |
| cart | gun | pan | sleigh | whisk |
| cheese | ham | peas | slide | witch |
| claw | hand | peg | snail | worm |
| club | harp | pie | snake | wreath |


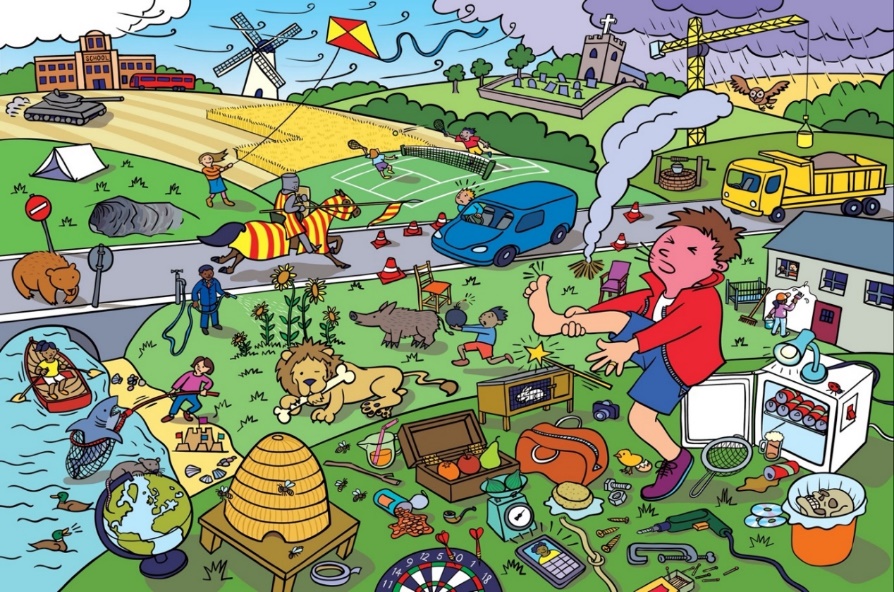


Sample example: There is a dartboard in the middle of the bottom line. The dart is in. dartboard twenty. The score. forty on the board. Middle to the right is. phoned. and it is ringing with a person trying to contact him. A rope is next to. in. next to it with some. three screws. There is a sponge and water by the side. There's a drill. that's on the ground. And some nails in between. I don't know what it is. So the. there's a mask of a. in the bin. And there was some. fridge with some cans in. inside of them. One of the cans. fallen out and is open. There's a lamp on the top of the fridge and it's on. The boy is holding his leg. I should imagine he's kicked something. other. but I don't know where he has kicked it. There was a wand that is thrown down. I don't know whether he has hurt himself with the wand. There was a rabbit hut in the middle of the picture. and a rabbit inside them. There was fruit inside a. I don't know what it is. but a fruit inside. apple or something like that. And there was a stick by the side of the. and a cup with some milk and. not milk. fruit juice. and a straw coming out. There was. beehive on the table with some bees on it. There is a globe in the corner of the picture. and a mouse on top of the globe. is just by the side with some ducks on it and a shark in an net. which is being pulled by a a boy. He made some sandcastles and some shells on the shore. Next to the shark is. rowing boat with a boy inside it rowing. There was some plants. which the man is hosing down. onto the. keeping them alive.

**Supplementary Figure 2.** Picture 2 of 4 used in the custom picture description task, along with a sample script of the patient’s description.

**Supplementary Table 2.** Target words embedded in Picture 2.

| Picture 2: 100 items | | | | |
| --- | --- | --- | --- | --- |
| bag | cone | hive | pear | smoke |
| beach | cot | hose | phone | sponge |
| bean | court | hutch | pin | stalk |
| bear | crane | jail | pipe | stick |
| bee | cross | jug | plant | straw |
| beer | dart | kite | rain | suit |
| bin | disc | knight | rat | switch |
| boar | drill | knot | ring | tank |
| bomb | duck | lamp | roof | tape |
| bone | ear | lens | scale | tent |
| box | fig | lion | school | tire |
| broom | foot | match | screen | tree |
| bug | fridge | mill | screw | truck |
| can | fruit | mouth | seat | van |
| cap | glass | net | shark | wall |
| cave | globe | nut | shell | wand |
| chair | grass | oar | shoe | well |
| chick | grave | owl | sieve | wheat |
| clamp | head | paint | sign | wind |
| coach | hinge | paw | skull | zip |

**
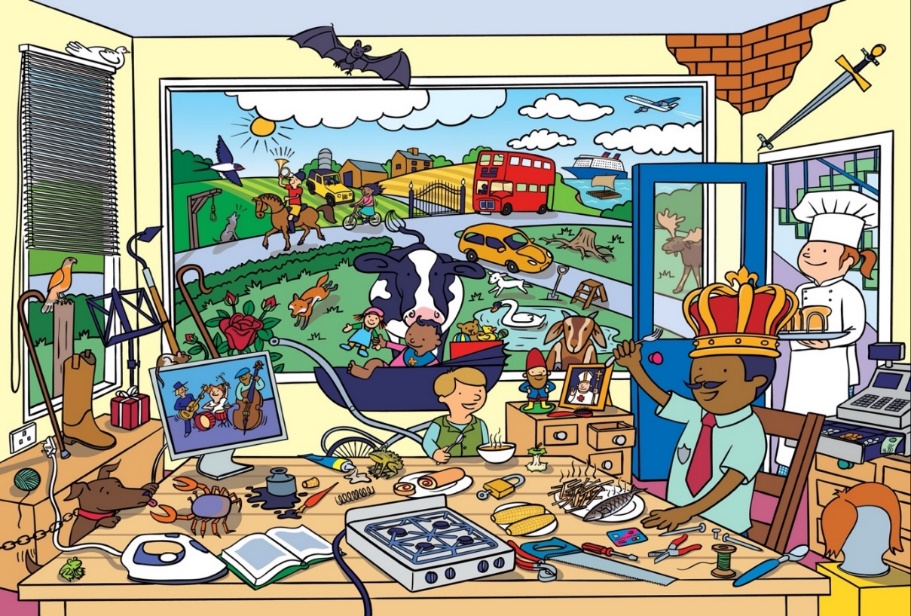
**

Sample example: There is a man acting as king with a shirt and tie on. He's acting as king for the boys that are sons which are here. He was eating a. fish and chips and a sweet corn. And there's a lock by the side of the lock by the side of the table. and the son is eating a bowl of soup. Somebody. either the man or the boy has eaten the apple. There was. on the table in the kitchen. there was an iron which is not plugged in. A tram. A book opened. and a gas cooker. on the table also. is the glue which is opened and some ink which is again open. and ink has fell on the table. Next to the ink is a pen. Old fashioned pen with ink around it. The dog is. has his head on the table and he's attempting to eat the crab. Next to the tables. is a table. not so. On there was a boot. A ball of wool and. present for whoever. Outside that there was a window and the bird in the. on the log outside. There was a. by the door. He coming in with an. toast. And the door is opened. The door is blue. and a red handle. and the glass is full. In the corner is a cash register that was. is. that is open and in that cash register is a. some money. Coins. There was a wig on the. in the right hand corner. And a spoon and the pliers. screwdriver. Nails. I don't know what that is. So on the wall. as. on the wall above the chef is a sword with the. hanging up and there is a picture on the background. In the picture there was a cow. a lamb. A swan. A horse. A deer. Horse and a fox. In the picture there was a cloud. An aeroplane and the sun is shining.

**Supplementary Figure 3.** Picture 3 of 4 used in the custom picture description task, along with a sample script of the patient’s description.

**Supplementary Table 3.** Target words embedded in Picture 3.

| Picture 3: 100 items | | | | |
| --- | --- | --- | --- | --- |
| arm | cloud | gnome | plane | stand |
| badge | core | goat | plate | step |
| band | cork | hawk | pliers | stove |
| bat | corn | hedge | plug | stump |
| bib | cow | hoe | pope | sun |
| bird | crab | hoof | pram | swan |
| blind | crook | horn | raft | sword |
| book | crown | ink | roll | thread |
| boot | dog | iron | root | tie |
| bowl | doll | jeep | rose | till |
| boy | door | knob | saw | toad |
| bricks | dove | lamb | scoop | toast |
| brush | farm | leg | ship | tools |
| bus | fish | lock | shorts | toys |
| cane | fork | man | skirt | vest |
| car | fox | moose | soup | wheel |
| card | frog | mouse | spade | wig |
| chain | gate | nail | spoon | wolf |
| chef | gift | noose | spring | wood |
| chips | glue | nose | stairs | wool |

**
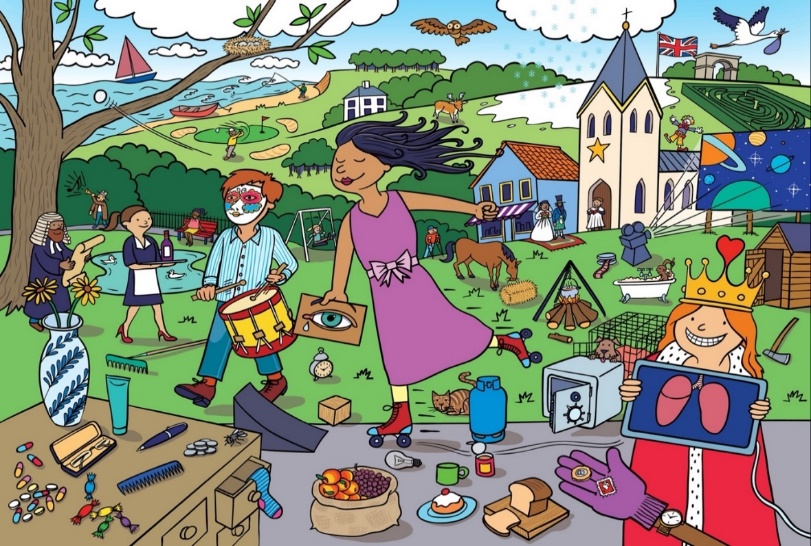
**

Sample example: Was a church in the front. and there's a bride and groom getting ready to go inside. The pastor is sitting outside the church and they are all on the cape. The clown is on the steeple and there was a star by the. on the front of the church. There's a maze in the background. nobody in it. and there's a stork going over with a bag in his mouth. The Union Jack is flying above the field. and there was an owl in the the sky. going up to his nest of four babies. In the. there is a sea on the. in the background. on top of which is a yacht. And there was a man fishing. There was an. a golf course in the way and the bloke is hitting a ball towards me. And he's going in to the tree. Just below that there. was there. was a bloke blowing a trumpet and a woman sitting on a bench. Next to the woman sitting on the bench was the child on the swing and next to it. in between the child on the swing and the groom and bride. was a youngster who's on crutches. There's a horse in the background. He's in. and there was a lock and a bowl of soup on a log. So there was a safe in the. on the bat by the Queen. And there was a glove. A stamp. And a coin on the club. I don't know who was come from. In the corner was a drawer. There was a comb on the top of the drawer and a pen and sunglasses. Pills and sweets. In the sock in the drawer was a sock which was opened. There. a vase on the top of it and some. and two flowers inside the vase. There was a rake on the ground which was in a. So there was a clock and a cat on the floor and a woman on roller skates.

**Supplementary Figure 4.** Picture 4 of 4 used in the custom picture description task, along with a sample script of the patient’s description.

**Supplementary Table 4.** Target words embedded in Picture 4.

| Picture 4: 100 items | | | | |
| --- | --- | --- | --- | --- |
| arch | coat | heart | pills | sock |
| axe | coins | heel | pot | sole |
| bath | comb | horse | pound | space |
| beard | crutch | house | priest | stamp |
| bench | deer | jeans | queen | star |
| block | drawers | judge | rake | stork |
| blouse | dress | kilt | ramp | sweet |
| boat | drum | lips | rod | swing |
| bow | eye | log | rug | tail |
| branch | face | lungs | sack | tear |
| bread | film | maid | safe | teeth |
| bride | fist | mask | sail | tile |
| bulb | flag | maze | sand | tin |
| bun | fly | mug | scroll | tray |
| cage | gas | neck | shed | tube |
| case | glove | nest | shirt | vase |
| cat | golf | note | shop | watch |
| church | grapes | park | skate | wave |
| clock | hair | peach | sleeve | wine |
| clown | hay | pen | snow | wing |

| **Supplementary Table 5. Participants’ demographic and clinical data** | | | | | |
| --- | --- | --- | --- | --- | --- |
| **ID** | **Gender** | **Age (years)** | **Education (years)** | **Time post stroke (years)** | **Lesion Volume (**$\boldsymbol{cm}^{\boldsymbol{3}}$**)** |
| 1 | M | 55 | 16 | 11 | 61.53 |
| 2 | M | 56 | 11 | 7 | 160.80 |
| 3 | M | 71 | 13 | 2 | 42.76 |
| 4 | M | 55 | 11 | 9 | 57.24 |
| 5 | M | 71 | 16 | 2 | 78.36 |
| 6 | F | 51 | 13 | 13 | 43.42 |
| 7 | M | 47 | 13 | 12 | 161.81 |
| 8 | F | 66 | 16 | 18 | 83.18 |
| 9 | M | 61 | 16 | 4 | 63.56 |
| 10 | M | 44 | 16 | 3 | 38.14 |
| 11 | F | 44 | 17 | 1 | 29.49 |
| 12 | F | 70 | 11 | 12 | 8.93 |
| 13 | M | 70 | 11 | 29 | 117.55 |
| 14 | M | 69 | 16 | 7 | 171.71 |
| 15 | M | 73 | 11 | 11 | 71.31 |
| 16 | F | 45 | 11 | 3 | 63.53 |
| 17 | F | 53 | 11 | 5 | 22.25 |
| 18 | F | 55 | 13 | 5 | 1.51 |
| 19 | M | 40 | 17 | 8 | 163.75 |
| 20 | M | 64 | 13 | 24 | 308.18 |
| 21 | M | 42 | 17 | 1 | 65.80 |
| 22 | M | 74 | 16 | 11 | 164.53 |
| 23 | M | 63 | 16 | 25 | 156.91 |
| 24 | M | 64 | 16 | 10 | 348.23 |
| 25 | M | 60 | 16 | 11 | 94.32 |
| 26 | F | 60 | 11 | 6 | 223.29 |
| 27 | M | 75 | 11 | 12 | 112.55 |
| 28 | F | 50 | 16 | 3 | 130.60 |
| 29 | M | 64 | 11 | 8 | 240.39 |
| 30 | M | 29 | 13 | 6 | 78.61 |
| 31 | F | 81 | 10 | 16 | 99.38 |
| 32 | M | 60 | 13 | 20 | 403.11 |
| 33 | M | 65 | 13 | 14 | 387.17 |
| 34 | M | 82 | 13 | 34 | 152.61 |
| 35 | M | 58 | 16 | 8 | 239.29 |
| 36 | M | 39 | 17 | 2 | 95.70 |

Lesion volume were estimated by the automated lesion identification method ^3^

| **Supplementary Table 6. Thirty-six patients’ standardised assessment and FLAT connected speech scores of custom picture description task.** | | | | | | | |
| --- | --- | --- | --- | --- | --- | --- | --- |
|  | CAT Spoken Comprehension | | CAT Speech Production | | FLAT scores of custom picture description | | |
|  | Words | Sentences | Naming | Picture Des | Bigram Fre | Bigram Col | Connectivity |
| 1 | **17** | **25** | **41** | **20** | **1973.69** | 15.01 | **0.47** |
| 2 | 26 | **19** | **13** | **14** | **2205.73** | 15.96 | **0.38** |
| 3 | **22** | 28 | **38** | **8** | 1058.86 | 9.06 | **0.51** |
| 4 | 30 | **18** | **26** | **8** | **1932.78** | 12.09 | **0.13** |
| 5 | 30 | 28 | **42** | 38 | 1269.23 | 10.96 | **0.68** |
| 6 | 26 | 28 | **38** | 40 | 1545.17 | 11.62 | **0.58** |
| 7 | 30 | **13** | **30** | **7** | **2375.66** | 13.97 | **0.34** |
| 8 | **22** | **16** | **30** | **19** | 1651.71 | 12.02 | **0.34** |
| 9 | **20** | **13** | **17** | **5** | **2409.99** | **21.43** | **0.44** |
| 10 | 28 | **18** | **34** | **20** | 1571.66 | 14.30 | **0.43** |
| 11 | 26 | **16** | **32** | **24** | 1600.73 | 13.45 | **0.52** |
| 12 | **25** | **25** | **35** | **26** | 1152.58 | 10.43 | **0.48** |
| 13 | 27 | **21** | **41** | **24** | **2277.86** | **17.99** | **0.57** |
| 14 | 30 | 30 | **29** | **13** | 1182.57 | 9.50 | **0.50** |
| 15 | 29 | **13** | **40** | **24** | 1773.48 | 15.75 | **0.51** |
| 16 | 29 | **23** | **41** | **22** | 1371.73 | 12.96 | **0.61** |
| 17 | 28 | **14** | **35** | **13** | 1620.55 | 13.10 | **0.47** |
| 18 | 30 | **24** | **39** | **32** | 1333.08 | 11.66 | **0.55** |
| 19 | 26 | 32 | **36** | **25** | 1228.18 | 11.83 | **0.55** |
| 20 | **24** | **21** | **29** | **11** | 1107.79 | 5.75 | **0.16** |
| 21 | 27 | **27** | **37** | **31** | 1386.84 | 10.85 | **0.57** |
| 22 | 28 | **22** | **38** | **13** | **2020.29** | 14.49 | **0.41** |
| 23 | 28 | **24** | **40** | **27** | 1264.18 | 10.41 | **0.65** |
| 24 | 29 | **18** | **40** | **18** | 562.60 | 1.72 | **0.51** |
| 25 | 29 | **24** | **40** | **13** | 204.75 | 3.19 | **0.43** |
| 26 | 26 | **23** | **43** | 35 | 1496.96 | 13.23 | **0.60** |
| 27 | 28 | 28 | **41** | 37 | 1344.78 | 10.97 | **0.69** |
| 28 | 30 | **25** | **39** | **20** | 1131.34 | 10.93 | **0.53** |
| 29 | 27 | **27** | **30** | **4** | **2847.93** | **20.82** | **0.37** |
| 30 | **22** | **18** | **33** | **32** | 1090.68 | 10.40 | **0.59** |
| 31 | 27 | **21** | **37** | **22** | 1641.05 | 12.41 | **0.56** |
| 32 | **24** | **20** | **21** | **1** | **3620.38** | **21.58** | **0.05** |
| 33 | 27 | **22** | **39** | **8** | **1810.27** | 13.71 | **0.15** |
| 34 | 29 | **27** | **43** | N/A | 1744.28 | 15.94 | **0.55** |
| 35 | 28 | **27** | **29** | N/A | **2061.85** | 14.57 | **0.26** |
| 36 | 29 | 32 | 48 | **11** | 1610.92 | 11.49 | **0.50** |
| cut off | 25 | 27 | 43 | 33 | 1804 | 16.52 | 0.7 |
| Cut-off score for CAT is sourced from the CAT manual^4^, while FLAT was obtained from 18 healthy participants completing identical composite picture tasks (see Supplementary Tables 7 and 8). Bold values indicate scores below cutoff: for FLAT, bold values in frequency and collocation are above average plus two standard deviations, while for connectivity, they are below average minus two standard deviations. Abbreviation: Picture Des- picture description; Bigram Fre- bigram frequency; Bigram Col- bigram collocation; FLAT- Frequency Language Analysis Tool. N/A values were replaced with the mean for subsequent statistical analyses. | | | | | | | |

**S2. Normative data:**

Individual FLAT scores of 18 healthy participants on the custom picture description tasks and their demographic data can be found in Supplementary Tables 7 and 8.

| **Supplementary Table 7. Healthy participants’ demographic characteristics** | | | | | |  |
| --- | --- | --- | --- | --- | --- | --- |
|  | Gender | Age (years) | Highest level Education | Handedness | First language |  |
| 1 | female | 69 | MA | right | English |  |
| 2 | male | 61 | BA | right | English |  |
| 3 | female | 79 | BA | right | English |  |
| 4 | male | 74 | MA | right | English |  |
| 5 | female | 80 | BA | right | English |  |
| 6 | female | 71 | MSc | right | English |  |
| 7 | male | 81 | BA | right | English |  |
| 8 | male | 63 | Phd | right | English |  |
| 9 | male | 72 | BA | right | English |  |
| 10 | female | 64 | BA | right | English |  |
| 11 | male | 63 | MA | right | English |  |
| 12 | female | 77 | highscool | right | English |  |
| 13 | female | 61 | MA | right | English |  |
| 14 | male | 66 | Phd | right | English |  |
| 15 | male | 61 | BA | irght | English |  |
| 16 | male | 52 | ----- | right | English |  |
| 17 | male | 63 | HNC | right | English |  |
| 18 | male | 58 | BA | right | English |  |

| **Supplementary Table 8. Healthy participants’ FLAT scores** | | | |
| --- | --- | --- | --- |
|  | Bigram Frequency | Bigram Collocation | Connectivity |
| 1 | 691.97 | 6.06 | 0.82 |
| 2 | 2293.52 | 21.12 | 0.70 |
| 3 | 1237.68 | 11.54 | 0.77 |
| 4 | 1368.81 | 12.19 | 0.77 |
| 5 | 1109.39 | 11.00 | 0.83 |
| 6 | 1044.59 | 10.23 | 0.80 |
| 7 | 763.82 | 7.29 | 0.83 |
| 8 | 763.81 | 7.55 | 0.83 |
| 9 | 972.13 | 8.75 | 0.76 |
| 10 | 1067.58 | 8.17 | 0.83 |
| 11 | 1045.17 | 10.19 | 0.78 |
| 12 | 858.12 | 8.41 | 0.79 |
| 13 | 1058.65 | 9.37 | 0.77 |
| 14 | 769.49 | 7.26 | 0.85 |
| 15 | 1273.34 | 11.20 | 0.78 |
| 16 | 1136.69 | 10.29 | 0.81 |
| 17 | 1218.42 | 10.99 | 0.78 |
| 18 | 999.89 | 8.87 | 0.88 |
| Mean (SD), range | 1092.94 (356.10), 691 - 2293 | 10.02 (3.25), 6 - 21 | 0.79 (0.04), 0.7-0.88 |

**S3. Parcellation of functional ROIs**

Supplementary Figure 5 shows the functional ROIs from the language network and the multiple demand (MD) network defined by Fedorenko et al.^5,6^ These ROIs were used as input masks for the Lesion Quantification Toolkit ^7^ with the AAL parcellation ^8^. Supplementary Table 9 lists all the parcels with corresponding proportion included in the analyses.


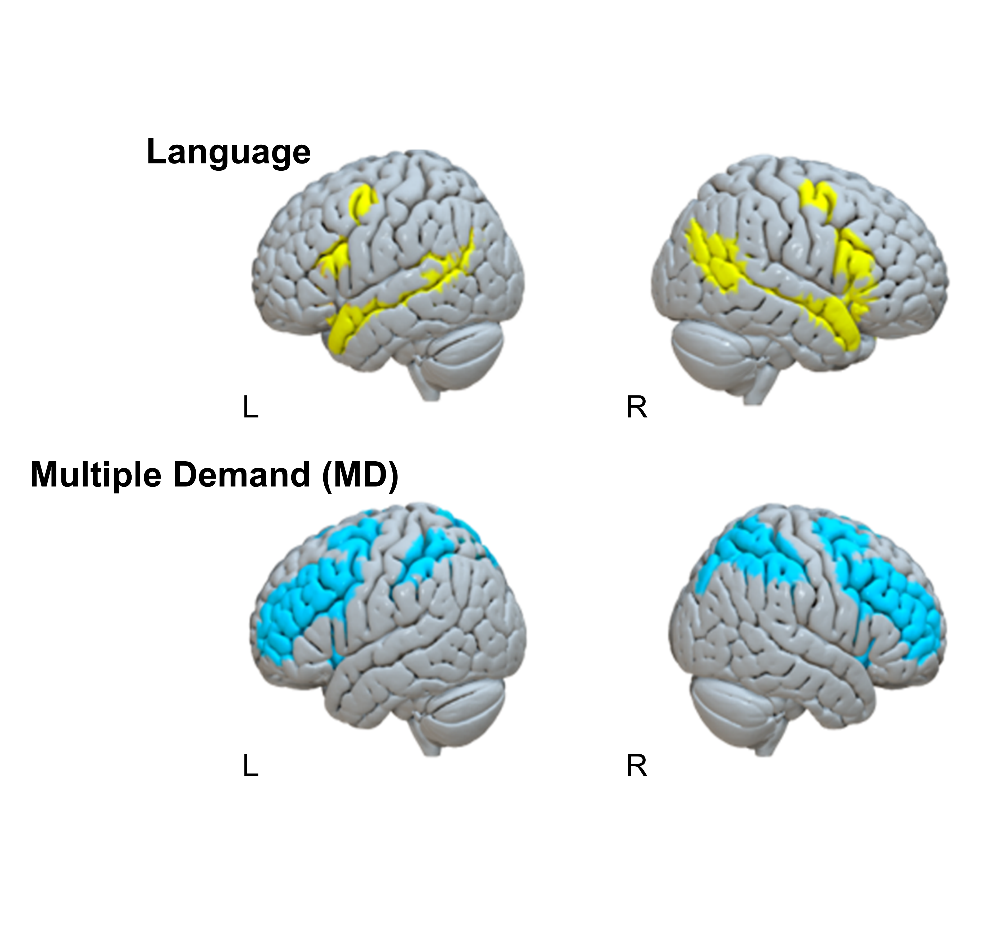


**Supplementary Figure 5.** Cortical surface rendering of two fROIs (rendered in SurfIce), language network and multiple demand (MD) network. L denotes the left hemisphere. R denotes the right hemisphere.

**Supplementary Table 9 – Language and MD networks ROIs and corresponding AAL parcels**

| Functional ROI mask | AAL parcels included | |
| --- | --- | --- |
| ​  Language ​ | ​  Precentral_L (15.14%), Precentral_R (10.09%)  ​  Frontal_Inf_Oper_L (30.23%), Frontal_Inf_Oper_R (31.64%) ​  Frontal_Inf_Tri_L (31.05%), Frontal_Inf_Tri_R  (28.98%)  Frontal_Inf_Orb_L(15.95%), Frontal_Inf_Orb_R  ​(14.49%)  Occipital_Mid_L (10.09%), Occipital_Mid_R  ​(15.36%)  SupraMarginal_L  ​(16.46%)  Angular_L (37.13%), Angular_R ​(26.61%)  Temporal_Sup_L (33.02%), Temporal_Sup_R  ​(38.55%)  Temporal_Pole_Sup_L (26.47%), Temporal_Pole_Sup_R (24.72%) ​  Temporal_Mid_L(65.57%), Temporal_Mid_R  (61.50%)​  Temporal_Pole_Mid_L (22.76%), Temporal_Pole_Mid_R ​(19.55%)  ​ | |
| ​  Multiple Demand (MD) ​ | | ​  Precentral_L (45.85%), Precentral_R (31.86%)  Frontal_Sup_L (26.95%), Frontal_Sup_R  ​(32.63%)  Frontal_Sup_Orb_L ​(11.81%)  Frontal_Mid_L (65.14%), Frontal_Mid_R  ​(64.24%)  Frontal_Mid_Orb_L (20.39%), Frontal_Mid_Orb_R ​(16.23%)  Frontal_Inf_Oper_L (52.53%), Frontal_Inf_Oper_R (54.30%) ​  Frontal_Inf_Tri_L (43.41%), Frontal_Inf_Tri_R  (32.61%)  Supp_Motor_Area_L (28.71%), Supp_Motor_Area_R  (20.83%)  Frontal_Sup_Medial_L  ​(15.80%)  Insula_L (26.79%), Insula_R (24.46%)  ​  Cingulum_Ant_L (14.20%), Cingulum_Ant_R ​(12.29%)  Cingulum_Mid_L (11.36%), Cingulum_Mid_R  ​(19.00%)  Cuneus_R  ​(23.37%)  Occipital_Sup_L (26.20%), Occipital_Sup_R  (38.08%)  Occipital_Mid_L (11.62%), Occipital_Mid_R  (10.72%)​  Postcentral_L (13.04%), Postcentral_R  ​(12.60%)  Parietal_Sup_L (82.65%), Parietal_Sup_R ​(80.60%)  Parietal_Inf_L (86.76%), Parietal_Inf_R (84.47%) ​  SupraMarginal_L (20.13%), SupraMarginal_R  (39.44%)​  Angular_L (18.02%), Angular_R  ​(28.60%)  Precuneus_L (41.05%), Precuneus_R (39.51%)​  ​ |

*Note: Functional ROIs of language-selective and domain-general network are defined by Fedorenko et al^5,6^. Parcels names are defined as the same in AAL* ^8^*.*

We included overlapping AAL regions when using functional regions of interest (fROIs), excluding only those parcels that comprised less than 10% of the total area. For Language, we excluded five small regions Frontal_Mid_L (1.29%), Insula_R (1.77%), Postcentral_L (0.19%), Temporal_Inf_L (1.3%), and SupraMargnial_R (5.6%). For the Multiple Demand (MD) network, we excluded eight regions: Putamen_L (0.48%), Cuneus_L (6.31%), Frontal_Inf_Orb_L (6.89%), Frontal_Inf_Orb_R (4.91%), Rolandic_Oper_L (2.21%), Rolandic_Oper_R (2.45%), Frontal_Sup_Orb_R (7.77%), and Frontal_Sup_Medial_R (8.10%). All region names follow AAL labels.

**S4. Materials for reproducibility**

Lesion quantification was conducted using the same script provided in Lesion Quantification Toolkit ^7^ in MATLAB. Python codes of analyse, structural connectivity data and behaviour excel files can be found in [UCL-NT/SNE-repo (github.com)](https://github.com/UCL-NT/SNE-repo). Patient raw data are available under restricted access and can be obtained by direct request to J.C.

**References**

1. Zimmerer VC, Newman L, Thomson R, Coleman M, Varley RA. Automated analysis of language production in aphasia and right-hemisphere damage: frequency and collocation strength. *Aphasiology*. 2018;32(11):1267-1283.

2. Zimmerer VC, Hardy CJD, Eastman J, et al. Automated profiling of spontaneous speech in primary progressive aphasia and behavioral-variant frontotemporal dementia: An approach based on usage-frequency. *Cortex*. 2020;133:103-119.

3. Seghier ML, Ramlackhansingh A, Crinion J, Leff AP, Price CJ. Lesion identification using unified segmentation-normalisation models and fuzzy clustering. *Neuroimage*. 2008;41(4):1253-1266.

4. Swinburn Kate. *Comprehensive Aphasia Test : CAT*. (Howard D, Porter Gillian, eds.). Psychology Press; 2004.

5. Fedorenko E, Duncan J, Kanwisher N. Language-Selective and Domain-General Regions Lie Side by Side within Broca’s Area. *Current Biology*. 2012;22(21):2059-2062.

6. Fedorenko E, Thompson-Schill SL. Reworking the language network. *Trends Cogn Sci*. 2014;18(3):120-126.

7. Griffis JC, Metcalf N V., Corbetta M, Shulman GL. Lesion Quantification Toolkit: A MATLAB software tool for estimating grey matter damage and white matter disconnections in patients with focal brain lesions. *Neuroimage Clin*. 2021;30:102639.

8. Tzourio-Mazoyer N, Landeau B, Papathanassiou D, et al. Automated Anatomical Labeling of Activations in SPM Using a Macroscopic Anatomical Parcellation of the MNI MRI Single-Subject Brain. *Neuroimage.* 2002; 15(1): 273-289.
